# A Rare Case of Non-Small Cell Lung Cancer with BRAF V600E Gene: Case Report and Literature Review

**DOI:** 10.7759/cureus.7055

**Published:** 2020-02-20

**Authors:** Dharti Patel, Ateeq Mubarik, Shilen Patel, Ali Vaziri, Salman Muddassir

**Affiliations:** 1 Internal Medicine, Oak Hill Hospital, Brooksville, USA; 2 Internal Medicine, Ascension St. Michael's Hospital, Stevens Point, USA; 3 Sleep Medicine, New York Sleep Disorder Center, Brooksville, USA; 4 Oncology, Oak Hill Hospital, Brooksville, USA; 5 Internal Medicine, Oak Hill Hospital, Broooksville, USA; 6 Internal Medicine, Hospital Corporation of America West Florida GME Consortium / Oak Hill Hospital, Brooksville, USA

**Keywords:** non-small cell lung cancer, braf mutation, immunotherapy, braf inhibitors

## Abstract

Non-small cell lung cancer is one of the leading causes of mortality in the United States. The BRAF mutation, which has been associated with malignant melanoma, has been documented in only 3.5-5% of the non-small cell lung cancer (NSCLC) patient population.The involvement of the BRAF mutation in NSCLC and the treatment for tumors with such mutations is still an evolving topic of interest, which is why more in depth information is warranted. We present a rare case of stage IV non-small cell lung adenocarcinoma, who presented first with a complicated pericardial effusion with evidence of malignant effusion. He had genetic testing done, revealing he had a positive BRAF V600E mutation. He was put on multiple chemotherapy regimens, but was most responsive to Vemurafenib. This case will shed light into the importance of the BRAF V600E gene and its importance in NSCLC for better prognosis value.

## Introduction

Lung cancer is one of the leading causes of mortality in the United States, with 90% of the lung cancers being non-small cell lung adenocarcinoma. BRAF mutations have been documented in only 3.5-5% of the non-small cell lung cancer (NSCLC) patients [1 ]. The occurrence of BRAF V600E mutations account for 50% of these cases, and the rest of BRAF mutations are non-V600E. Various other gene markers that have been associated with non-small cell lung cancer include EGFR, VEGF, ALK-EML4 mutations. We present a case of stage IV non-small cell lung adenocarcinoma, who presented first with a complicated pericardial effusion with evidence of malignant effusion. He was found to have PD-L1 90%, G360, with a positive BRAF V600E. He had multiple chemotherapy regimens, but was most responsive to Vemurafenib. Currently, he is still on this regimen, and is tolerating it well.

## Case presentation

**Figure 1:**
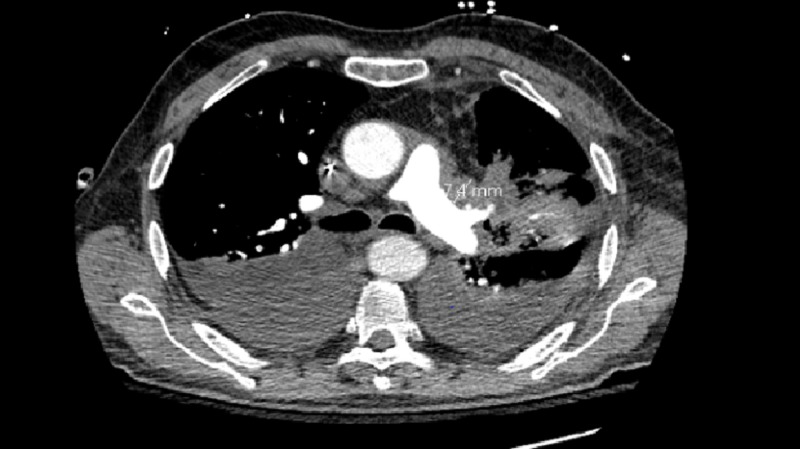
CT scan showing 7.4 mm mass in left lingula Bilateral pleural effusions, diffuse pleural thickening,and bilateral lung base air space opacity,soft tissue density in bilateral hilar region and bilateral bronchus wall thickening more prominent on the left, pleural effusion  given slight loculated appearance especially on the left side Presence of diffuse osteoblastic metastatic disease

The patient is a 69-year-old male with a past medical history of dyslipidemia, brain aneurysm status post repair, benign prostatic hyperplasia, thyroiditis who was seen inpatient as he presented with shortness of breath, difficulty swallowing, cough, fatigue, unintentional weight loss of 12 pounds in the last five weeks. Upon his first admission to the hospital, his labs were: WBC: 9.8x 10^3^/uL , RBC of 4.42x10^6^/uL, hemoglobin of 13.3 g/dl, hematocrit of 41.4%, MCV of 93.7fl, MCH: 30.1 pg, MCHC of 32.1 g/dl, RDW of 42.1 fl, and a differential showing increase in lymphocytes of 11.1%. His chemistries showed a sodium of 144 mmol/l, potassium of 4.1 mmol/l, magnesium of 2.3 mg/dl, phosphate of 3.3 mg/dl, AST of 20 U/l, ALT of 45 U/l, creatinine of 1 mg/dl, albumin of 3.4 g/dl, and CA 19-9 of <1.4. Initial imaging on CT chest was found to have a mild pericardial effusion with bilateral pleural effusions, multiple nonspecific small minimally prominent bilateral cervical lymph nodes with small-to-moderate confluent consolidation in the posterior central lingula extending to the hilum, with the impression that malignancy could not be excluded (figure 1). He then proceeded to undergo a thoracentesis. He subsequently developed fairly rapid reaccumulation of fluid and was concerned about a potential underlying empyema and therefore, transferred to a facility with thoracic surgery specialty. At our facility, cardiothoracic surgery performed a pericardial window. 

The final pathology of 600ccs of serosanguineous fluid was suggestive of poorly differentiated malignancy, with stains positive for CK7, TTF1 and NAPSIN A, suggestive of metastatic stage IV adenocarcinoma of the lung. Additional stains showed that he had negative PAX8 and CD68, and was also negative for ALK mutations. An additional gene study was done which showed positive BRAF V600E mutation. He eventually had to have placement of a pleural catheter on the left side, since there was continuous reaccumulation of pleural fluid. 

He had another episode of pleural effusion on the right side, had a right side thoracocentesis, and developed a pneumothorax for which he had to have a chest tube inserted. His pneumothorax improved, and he was discharged home and instructed by his oncologist to start BRAF inhibitors and MEK inhibitors. While he was following outpatient with oncology, over time, his chemotherapy regimens included being started on palliative Carboplatin, Alimenta with Keytruda, then Tafinlar/Mekinist combination, and then finally on Vemurafenib. He stated the BRAF inhibitors did help him. He currently follows outpatient with oncology. 

## Discussion

Most lung cancers (85%-90%) are found to be non-small cell lung cancer [2]. Various genetic markers that have been associated with non-small cell lung cancer include EGFR mutations, EML-ALK fusions, ROS1, MET, KRAS, HER2, and less commonly BRAF [3]. The BRAF gene is an oncogene that is a part of the RAS/MPK pathway that regulates cell growth and division. Overexpression of this gene leads to increased proliferation of cells, leading to malignancy.The gene is more associated with melanoma, metastatic colorectal cancer, and papillary thyroid cancer, with the most common frequency in melanoma, of 50% [4-6]. BRAF mutations were found in 1-3% of NSCLCs [7]. BRAF- V600E mutations occur predominantly in females, smokers, and also has a pathology of adenocarcinoma [1,8]. Our patient was a male, nonsmoker, with the histology of adenocarcinoma. The analysis of the BRAF V600E mutation has helped identify two medications that have shed some light on how to help improve patient outcomes. 

Vemurafenib is a potent inhibitor of the BRAFV600E mutation, with minimal antitumor effects against cells with wild-type BRAF [4,9,10]. A phase 1 trial established the most efficacious dose of 960 mg twice daily for the best tumor response (10). Vemurafenib was associated with a relative reduction of 63% in the risk of death and 74% in the risk of tumor progression, as compared with dacarbazine (p<0.001 for both comparisons). Studies have shown that the median progression-free survival (PFS) was 5.3 months in the vemurafenib group and 1.6 months in the dacarbazine group [11 ]. A case report of a male patient with BRAF V600E lung adenocarcinoma showed a good clinical and metabolic response when treated with vemurafenib [12]. 

Another drug that has been extensively studied is Dabrafenib. It is a reversible, potent and selective inhibitor of BRAF V600E kinase activity, which is known to have competitive inhibition against adenosine triphosphate. The dose of 150 mg orally twice a day was selected based on the pharmacokinetics and the effects of dabrafenib on a molecular biomarker target (tumor pERK inhibition), FDG-PET metabolic uptake, and the safety profile [13]. In another multicenter, open-label, phase 2 trial, there was clinical evidence that dabrafenib showed clinical activity in subjects with BRAF V600E mutation positive melanoma with brain metastases who never received dabrafenib (cohort A) as well as those who previously received local therapy (cohort B) [14]. Furthermore, dabrafenib has shown a response rate of 59% in a phase 2 trial for determining the efficacy in patients with BRAF mutation positive metastatic melanoma [15].

In another phase III study, comparing dacarbazine vs dabrafenib patients with unresectable or metastatic BRAF V600E mutation positive melanoma, treatment with dabrafenib resulted in significant improvement in PFS, response rate, and duration of response over dacarbazine. Dacarbazine is an alkylating agent that inhibits DNA synthesis and has been used in metastatic malignant melanoma. The progression free survival was 5.1 months for dabrafenib and 2.7 months for dacarbazine [16]. 

Common adverse events (AE) associated with BRAF inhibitors include arthralgia, rash, fatigue, alopecia, photosensitivity, nausea and diarrhea, headaches, fevers, joint pain, keratoacanthoma or squamous-cell carcinoma and even in rare cases new melanomas [17]. BRAF-inhibitor induced cutaneous squamous-cell carcinoma is a result of the paradoxical RAF inhibitor-mediated activation of the RAS signaling pathway in BRAF wild-type cells [18].

## Conclusions

This case provides further insight into the importance of genetic testing on metastatic non-small cell lung cancer with the positive testing of BRAFV600E. Despite lung cancer being a leading cause of mortality, treatment outcomes for those who have this rare gene, have been shown to have benefited from newer drug modalities including the BRAF inhibitors, even second-line options including immunotherapy. More research with prospective studies is needed to establish efficacy in new regimens in BRAF positive mutations with non-small cell metastatic adenocarcinoma. 
